# Moderate Highland Barley Intake Affects Anti-Fatigue Capacity in Mice via Metabolism, Anti-Oxidative Effects and Gut Microbiota

**DOI:** 10.3390/nu17040733

**Published:** 2025-02-19

**Authors:** Liangxing Zhao, Qingyu Zhao, Sameh Sharafeldin, Luman Sang, Chao Wang, Yong Xue, Qun Shen

**Affiliations:** 1 College of Food Science and Nutritional Engineering, China Agricultural University, Beijing 100083, China; zhaoliangxing23@163.com (L.Z.); zhaoqingyu@cau.edu.cn (Q.Z.); samehgamil@agr.dmu.edu.eg (S.S.); sang18438616120@163.com (L.S.); iamllacc1999@126.com (C.W.); xueyong@cau.edu.cn (Y.X.); 2 National Grain Industry (Highland Barley Deep Processing) Technology Innovation Center, Beijing 100083, China; 3 National Grain and Oil Standards Research Verification and Testing Center, Beijing 100083, China; 4 Department of Food and Dairy Sciences and Technology, Faculty of Agriculture, Damanhour University, Damanhour 22516, Egypt

**Keywords:** highland barley, anti-fatigue, intake level, gut microbiota

## Abstract

Objectives: this study aimed to explore the effects of different intake levels (20–80%) of highland barley on the anti-fatigue capacity of ICR mice, focusing on energy metabolism, metabolite accumulation, oxidative stress, and changes in the gut microbiota. Methods: male ICR mice were assigned to five groups: control (normal diet) and four experimental groups with highland barley supplementation at 20%, 40%, 60%, and 80% of total dietary energy. Anti-fatigue performance was assessed by behavioral experiments (rotarod, running, and exhaustive swimming tests), biochemical markers, and gut microbiota analysis. Results: the results showed that moderate supplementation (20%) significantly enhanced exercise endurance and anti-fatigue capacity, as evidenced by increased liver glycogen (134.48%), muscle glycogen (87.75%), ATP content (92.07%), Na^+^-K^+^-ATPase activity (48.39%), and antioxidant enzyme activities (superoxide dismutase (103.31%), catalase (87.75%), glutathione peroxidase (81.14%). Post-exercise accumulation of blood lactate, quadriceps muscle lactate, serum urea nitrogen, and the oxidative stress marker malondialdehyde was significantly reduced, with differences of 31.52%, 21.83%, 21.72%, and 33.76%, respectively. Additionally, 20% supplementation promoted the growth of beneficial gut microbiota associated with anti-fatigue effects, including *unclassified_f_Lachnospiraceae*, *g_norank_f_Peptococcaceae*, *Lachnospiraceae NK4A136*, *Colidextribacter*, and *Turicibacter*. However, when intake reached 60% or more, anti-fatigue effects diminished, with decreased antioxidant enzyme activity, increased accumulation of metabolic waste, and a rise in potentially harmful microbiota (*Allobaculum*, *Desulfovibrio*, and *norank_f_norank_o_RF39*). Conclusions: moderate highland barley supplementation (20% of total dietary energy) enhances anti-fatigue capacity, while excessive intake (≥60%) may have adverse effects.

## 1. Introduction

Exercise-induced fatigue is a complex physiological response involving multiple factors, including energy depletion, metabolite accumulation, oxidative stress, immune responses, and gut microbiota dynamics [1,2]. During exercise, the depletion of energy reserves such as glycogen, liver glycogen (HG), muscle glycogen (MG), phosphocreatine, and adenosine triphosphate (ATP) leads to reduced muscle-contraction efficiency and limits exercise endurance [3,4]. Furthermore, impairments in ATP production and utilization contribute to the accumulation of metabolic byproducts [5]. In addition, exercise generates metabolic byproducts such as lactic acid (LD), blood lactate (BLA), serum urea nitrogen (BUN), and hydrogen ions, which may cause acidosis, disrupt normal muscle function and ion balance, and further exacerbate fatigue [6]. During exercise, increased oxygen demand accelerates metabolism, leading to the release of reactive oxygen species (ROS) from the mitochondria [7]. While a certain level of ROS is essential for normal muscle function [8,9], excessive ROS production can impair muscle contraction and disrupt energy metabolism, ultimately contributing to fatigue [10,11]. Prolonged exercise induces an immune response, elevating pro-inflammatory cytokines and activating immune cells, which can cause muscle damage, hinder recovery, and exacerbate fatigue [12]. During the onset of fatigue, lactate accumulation and energy depletion are closely linked to the metabolism of the gut microbiota. Meanwhile, dysbiosis caused by an increase in pathogenic bacteria and a decrease in beneficial bacteria not only exacerbates oxidative stress and inflammatory responses but also compromises gut barrier function, further aggravating fatigue [2].

At present, the concept of “homology of medicine and food” is considered a feasible approach to ensuring nutritional safety and promoting public health [13]. Previous studies have reported that supplementation with carbohydrates can help meet the physiological demands of glucose homeostasis, provide energy to the body, and enhance glycogen storage, thereby alleviating fatigue during exercise [14,15]. Additionally, β-glucan exerts anti-fatigue effects by increasing glycogen stores in the body [16]. Additionally, polysaccharide supplementation has been shown to restore fatigue-related imbalances in the gut microbiome, for example, reversing the decline in the Firmicutes-to-Bacteroidetes ratio [17]. Furthermore, an increased abundance of *Turicibacter*, *unclassified Lachnospiraceae*, and *Lachnospiraceae NK4A136* in the gut microbiota has been linked to improved muscle function and reduced fatigue through their support of energy metabolism and enhanced nutrient catabolism [18,19,20,21]. Dietary fiber supports gut health by facilitating the excretion of metabolic waste such as BUN and promoting a steady release of blood glucose, thereby reducing fatigue associated with metabolic byproducts [22]. On the other hand, proteins contribute to fatigue resistance by supplying essential nutrients such as branched-chain amino acids (BCAAs), arginine, and other amino acids that aid in post-exercise muscle recovery, promote liver and muscle glycogen synthesis, and enhance energy metabolism [23]. Specifically, γ-aminobutyric acid (GABA) has been shown in human trials to reduce fatigue and improve daily work performance [24]. Polyphenolic compounds mitigate oxidative stress by neutralizing ROS generated during strenuous exercise [25]. Furthermore, minerals also play a role in fatigue resistance. Vitamins and minerals such as iron, magnesium, and zinc are involved in key metabolic pathways that support essential cellular functions. By promoting energy metabolism, enhancing oxygen transport, and maintaining neuromuscular function, these micronutrients help sustain normal physiological activity and contribute to fatigue reduction [26].

Notably, studies have shown that dietary supplementation with oats (21.44 g/kg/day) can enhance swimming endurance and increase liver glycogen storage in mice [27]. This effect is attributed to the β-glucan in oats, which exerts anti-fatigue effects by boosting glycogen reserves in the body, thereby contributing to fatigue resistance [16]. Similarly, highland barley (*Hordeum vulgare* L.), which is rich in β-glucan, also contains bioactive compounds such as γ-aminobutyric acid, anthocyanins, and polyphenols [28]. Furthermore, according to the ancient Chinese medical book *Bencao Shiyi*, “Highland barley promotes internal smoothness and comfort, enhances essence and strength, boosts physical endurance and vitality, eliminates internal dampness, and alleviates or treats diarrhea”. The phrases “enhances essence and strength” and “boosts physical endurance and vitality” can be interpreted as referring to effects on the body’s anti-fatigue capabilities. Zhang et al. investigated the effects of five different doses (0.2, 0.4, 0.8, 1.6, and 2.4 g/kg·bw) of highland barley chlorophyll on the anti-fatigue capacities of mice using ladder-climbing and exhaustive swimming tests. The study demonstrated that, regardless of the dose, highland barley chlorophyll significantly increased climbing time and weight-bearing swimming endurance, resulting in a dose-dependent improvement in anti-fatigue performance [29]. Despite these findings for highland barley chlorophyll, there is limited evidence regarding whether whole-grain highland barley exhibits similar anti-fatigue properties. Given its content of bioactive compounds known for their anti-fatigue effects [14,15,16,22], this study aims to investigate the potential anti-fatigue properties of whole-grain highland barley and to determine the intake dose that elicits the most significant anti-fatigue effects, as assessed through physiological and biochemical markers associated with fatigue mitigation. Our hypothesis posits that whole-grain highland barley exerts anti-fatigue effects through the modulation of oxidative stress, enhancement of energy metabolism, and remodeling of the composition of the gut microbiota, thereby contributing to the alleviation of fatigue-related physiological responses.

This study investigates the effects of graded dietary-inclusion levels of highland barley, specifically, 20%, 40%, 60%, and 80% incorporation into feed, on energy metabolism, accumulation of metabolic byproducts, oxidative-stress biomarkers, and composition of the gut microbiota in ICR mice subjected to exercise. By focusing on whole-food interventions rather than isolated compounds, this approach aligns more closely with human dietary practices, while the exploration of varying intake levels aims to identify the optimal dosage for maximizing the health-promoting properties of highland barley.

## 2. Materials and Methods

### 2.1. Determination of Basic Nutritional Components in Highland Barley

The raw “Zangqing No. 3” highland barley flour was provided by Shanxi Dongfang Liang Life Science Technology Co., Ltd. (Datong, China). Its protein, fat, dietary fiber, crude polysaccharides, β-glucan, total polyphenols, ash, and moisture content were determined using the methods specified in the AACC standards: 46–30.01, 30–10.01, 32–07.01, enzymatic method, 32–23.01, 32–40.01, 08–01.01, and 44–15.02, respectively. The amino acid composition was analyzed using high-performance liquid chromatography (HPLC). Flavonoids, alkaloids, and other small molecules in highland barley were determined using the U3000 Ultra High-Performance Liquid Chromatography System (Thermo Fisher Scientific, Waltham, MA, USA) and the Thermo Q Exactive Plus mass spectrometer (Thermo Fisher Scientific, Waltham, MA, USA) (Table 1).

### 2.2. Animal Experiment Protocol

Male ICR mice (4 weeks old, 26–28 g) were purchased from Beijing Vital River Laboratory Animal Technology Co., Ltd. (Beijing, China) (License: SCXK (J) 2021-0006). All mice were pathogen-free and housed under controlled conditions (22 ± 1 °C; 12-h light/dark cycle; 55 ± 10% humidity) with free access to food and water. Food intake and body weight were recorded weekly.

All experimental procedures were conducted following the National Research Council guidelines and were approved by the Animal Care Committee of China Agricultural University (Approval No: AW12011202-4-1, approval date: 25 October 2021).

To determine the optimal dosage of highland barley supplementation, we referred to studies on whole-grain foods such as oats [27] and beans [30], as well as research on dosage conversion between animal and human studies [31]. Based on these references, we designed five experimental groups with varying supplementation levels to systematically evaluate the dose-dependent effects. Additionally, based on the relevant anti-fatigue research literature and a power calculation, eight mice were assigned to each group [16,32,33,34,35]. The details are as follows: after a one-week acclimatization period, the mice were randomly assigned to five groups: (1) normal diet (NC, *n* = 8) (AIN-93M); (2) normal diet supplemented with 20% highland barley powder (HB20, *n* = 8); (3) normal diet supplemented with 40% highland barley powder (HB40, *n* = 8); (4) normal diet supplemented with 60% highland barley powder (HB60, *n* = 8); (5) normal diet supplemented with 80% highland barley powder (HB80, *n* = 8).

The experimental diets were purchased from Changzhou Shu Yi Shu Er Biotechnology Co., Ltd. (Changzhou, China). The contribution of each macronutrient to the caloric density was consistent across the NC, HB20, HB40, HB60, and HB80 diets (Table S1). The composition of the diets is provided in Table S2.

**Figure 1:**
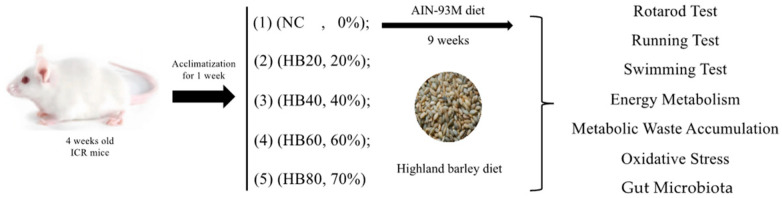
Experimental Design. Note: (1) normal diet (NC, *n* = 8) (AIN-93M); (2) normal diet supplemented with 20% highland barley powder (HB20, *n* = 8); (3) normal diet supplemented with 40% highland barley powder (HB40, *n* = 8); (4) normal diet supplemented with 60% highland barley powder (HB60, *n* = 8); (5) normal diet supplemented with 80% highland barley powder (HB80, *n* = 8).

From weeks 9 to 12, the mice underwent anti-fatigue tests, including the rotarod, treadmill, and exhaustive swimming tests (Figure 1).

### 2.3. Anti-Fatigue Experiment

- Rotarod Test

The Rotarod test was performed with modifications according to the method described by Li et al. [36]. In week 9, the first two days were used for acclimatization, and the formal experiment was conducted on day 3. The mice were placed on a rotarod set to 35 rpm; the total duration of the test was 500 s. The time taken for each mouse to fall off the rod was recorded. If a mouse did not fall off within 500 s, a time of 500 s was recorded as the exhaustion time. Three parallel tests were conducted. After each test, the rod was wiped with 75% alcohol and allowed to dry before the next trial.

- Running Test

After a 7-day rest following the Rotarod test, the mice underwent the treadmill endurance-running experiment. The method was slightly modified based on the protocol described by Seiler et al. [37].

The first 5 days were dedicated to the adaptation phase: on the first day, the speed of the conveyor belt on the running platform was set at 10 m/min and the inclination angle of the conveyor belt was 0°. The mice were gently placed on the track for a 5-min running adaptation. On the second day, the speed was set to 10 m/min for 5 min, then increased to 12 m/min for another 5 min. On the third day, the speed was set to 12 m/min for 5 min, then to 14 m/min for 5 min. On the fourth day, the speed was set to 14 m/min for 5 min, then increased to 16 m/min for 5 min more. On the fifth day, the speed was set to 14 m/min, 16 m/min, and 18 m/min for 4 min each.

On day 6, the testing phase began. The initial treadmill speed was set at 10 m/min, and the mice were placed on the treadmill. The speed was gradually increased by 2 m/min every 2 min until it reached 20 m/min. The test continued until the mice ran to exhaustion, defined as running for 120 min at 20 m/min. Mice were considered exhausted when they received five 0.5 mA electrical shocks within 5 s. After each trial, the treadmill was wiped with 75% alcohol and allowed to dry before the next trial.

- Exhaustive Swimming Test

After a 7-day rest following the treadmill experiment, the mice underwent the exhaustive swimming test. This test was performed with modifications based on the method described by Guo et al. [38]. A lead weight equivalent to 5% of each mouse’s body weight was attached to the base of its tail. The mice were placed in a swimming tank with a diameter of 110 mm and a height of 300 mm that had been filled with water to a depth of approximately 30 cm. The water temperature was maintained at around 20 °C. The test continued until the mouse sank completely to the bottom of the tank and did not resurface within 8 s. The time to exhaustion was recorded as the endpoint of the test.

### 2.4. Measurement of Anti-Fatigue-Related Indicators

Immediately after the exhaustive swimming test, blood was collected from the heart using a disposable syringe for analysis of hemoglobin (HGB) levels using an automated veterinary blood-cell analyzer (Mindray DC-5180, Mindray Bio-Medical Electronics Co., Ltd., Shenzhen, China). Additional blood samples were collected and were centrifuged at 3000 rpm for 15 min to obtain serum. The liver and quadriceps femoris muscles were then dissected and weighed. Organ indices were calculated using the following formula. The serum and tissue samples were stored at −80 °C until analysis of the relevant indicators.

Organ Index = Organ Weight (mg)/Body Weight (g).

### 2.5. Determination of Biochemical Indexes

Protein concentrations were determined using a BCA protein assay kit (Solarbio, Beijing, China). BLA, HGB, ATP content, NA^+^-K^+^-ATPase, HG, MG, BUN, and LD levels were measured using kits from the Nanjing Jiancheng Bioengineering Institute (Nanjing, China) according to the protocols of the manufacturer. Additionally, the levels of SOD, MDA, GSH-Px, and CAT in liver and muscle samples were measured using the same kits.

### 2.6. 16S rRNA Sequencing

The gut microbiota were analyzed as described previously [39]. Fecal samples were collected under sterile conditions, frozen in liquid nitrogen, and stored at −80 °C. Genomic DNA was extracted from the microbiota using the E.Z.N.A.^®^ Soil DNA Kit (Omega Bio-Tek, Norcross, GA, USA) and assessed for quality and concentration using a 1% agarose gel and NanoDrop 2000 UV-Vis spectrophotometer (Thermo Scientific, Wilmington, DE, USA). The V3–V4 region of the bacterial 16S rRNA gene was amplified using the primers 338F/806R (338F: 5′-ACTCCTACGGAGGCAGCAGCAGCAG-3′; 806R: 5′-GGACTACHVGGTWTCTAAT-3′). PCR products were purified with the AxyPrep DNA Gel Extraction Kit (Axygen Biosciences, Union City, CA, USA) and quantified using a Quantus™ Fluorometer (Promega, Madison, WI, USA). The purified amplicons were sequenced using the Illumina MiSeq PE 300 platform (Illumina, San Diego, CA, USA) with paired-end sequencing.

Quality screening and filtering of the raw 16S rRNA sequencing reads were performed using QIIME, and this step was followed by multiplexing, quality-filtering, and merging using Flash version 1.2.7. Operational taxonomic units (OTUs) were clustered at 97% similarity using UPARSE version 7.1, and chimeric sequences were removed. Representative sequences for each OTU were classified using the RDP Classifier with a confidence threshold of 0.7 against the 16S rRNA database. Diversity analysis included α-diversity metrics such as Shannon and Simpson indices, calculated using Mothur, and β-diversity, assessed via principal coordinates analysis (PCoA). Statistical significance in relative abundance between groups was determined using the Wilcoxon rank sum test. Linear discriminant analysis effect size (LEfSe) and linear discriminant analysis (LDA) (threshold = 3) were applied to analyze the alterations in the composition of the gut microbiota across the experimental groups.

### 2.7. Data Processing and Statistical Analysis

Statistical results are expressed as mean ± SEM (standard error of the mean). One-way analysis of variance (ANOVA) was used to perform multiple comparisons between the experimental groups (NC, HB20, HB40, HB60, and HB80). Specifically, biochemical indexes such as blood lactate (BLA), hemoglobin (HGB), superoxide dismutase (SOD), and other related measures were compared across groups. Differences between the groups for daily food intake and other measures within the same week were also analyzed. To further identify pairwise differences between the groups, Duncan’s post-hoc test was applied. *p*-values < 0.05 were considered statistically significant. Statistical computations were performed using SPSS version 22.0 (IBM Corporation, Chicago, IL, USA), and graphs were generated using GraphPad Prism version 9.0.1 software.

## 3. Results

### 3.1. Effects of Different Levels of Highland Barley Intake on Growth and Exercise Endurance in Mice

**Figure 2:**
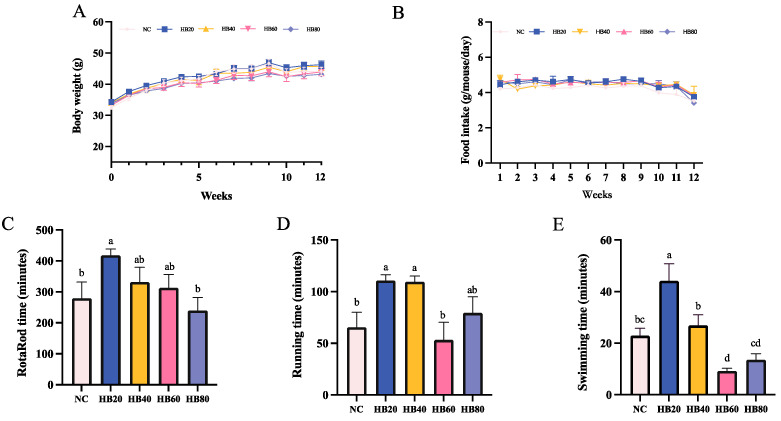
Effect of highland barley supplementation on body weight. (**A**), food intake (g/mouse/day) (**B**), fatigue measured by time to exhaustion in the rotarod test (**C**), fatigue measured by time to exhaustion in the treadmill test (**D**), and fatigue measured by time to exhaustion in the swimming test (**E**) in mice. Note: In (**A**,**B**), statistical comparisons were conducted between groups within the same week, and no significant differences were observed. Bars with different letters (e.g., a, b, c, d) indicate significant differences at *p* < 0.05. Bars without letters indicate no significant difference.

Changes in body weight reflect the impact of highland barley supplementation on the health status of mice. The body weight of mice in all groups gradually increased, with no significant differences in body weight or food intake across groups (Figure 2A). There were no significant differences in organ indices between groups (Figure 2B). No abnormal behavior or mortality were observed throughout the study, indicating that the increased highland barley intake did not adversely affect the growth or health of the mice.

#### 3.1.1. Rotarod Test

The rotarod endurance time for the HB20 group (417.79 ± 20.24 min) was significantly longer than that of the NC group (279 ± 52.83 min) (*p* < 0.05), with a positive difference of about 49.75%. However, although no supplementation levels below 20% were tested, the positive effect of 20% highland barley supplementation on endurance was diminished with higher doses. Specifically, when the supplementation level reached 60% or more, an adverse effect on endurance was observed, as evidenced by a gradual decrease in rotarod endurance time. The HB80 group (239.35 ± 20.24 min) had a rotarod time that was approximately 14.21% shorter than that of the NC group (Figure 2C). This suggests that 20% highland barley supplementation has the potential to enhance anti-fatigue capacity, while further increases in highland barley intake do not provide additional benefits and may reduce endurance.

#### 3.1.2. Running Test

Compared to the NC group (65.40 ± 14.71 min), the HB20 group (110.85 ± 5.63 min) and HB40 group (109.51 ± 5.60 min) had significantly longer treadmill endurance times: approximately 69.51% and 67.45% longer, respectively (*p* < 0.05). However, when the highland barley content was increased to 60% (HB60 group), the treadmill endurance time (53.19 ± 17.15 min) was significantly shorter than that of the HB20 group: about 52.02% shorter (*p* < 0.05). This suggests that a 60% highland barley diet negatively affects exercise endurance (Figure 2D).

#### 3.1.3. Swimming Test

Relative to the NC group (22.90 ± 2.94 min), the HB20 group (44.13 ± 6.67 min) exhibited a significantly increased swimming time to exhaustion (*p* < 0.05). However, as the highland barley intake increased, the swimming time to exhaustion gradually decreased. Specifically, the HB40 group (26.88 ± 4.09 min), HB60 group (9.20 ± 1.06 min), and HB80 group (13.59 ± 2.33 min) had swimming times to exhaustion that were significantly shorter than that of the HB20 group, by 39.08%, 79.15%, and 69.21%, respectively (*p* < 0.05). Additionally, the HB60 and HB80 groups showed significantly shorter times to exhaustion than the NC group, by 59.83% and 80% (*p* < 0.05), respectively (Figure 2E).

The results from all three anti-fatigue behavioral tests suggest that moderate supplementation with highland barley (20%) enhances anti-fatigue capacity, while higher doses (60% and 80%) may have a negative impact on endurance in mice.

### 3.2. Effects of Different Levels of Highland Barley Intake on the Organ Index of Mice

**Figure 3:**
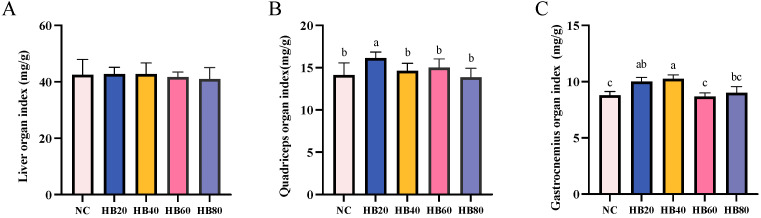
Effects of highland barley supplementation on the organ indices of the liver (**A**), quadriceps (**B**), and gastrocnemius (**C**) in mice. Bars with different letters (a, b, c) indicate significant differences at *p* < 0.05. Note: No significant differences were observed among groups in (**A**). Bars with different letters (e.g., a, b, c) indicate significant differences at *p* < 0.05. Bars without letters indicate no significant difference.

Skeletal muscles play an essential role in metabolism and exercise endurance. The organ index provides an accurate reflection of the weight of a specific organ as a proportion of the total body weight. The skeletal muscle organ index is a key indicator of muscle health and function [40]. In the HB20 group, the quadriceps (16.13 ± 0.25) and gastrocnemius (10.02 ± 0.36) muscle organ indices were 14.03% and 13.85% higher, respectively, compared to those of the NC group (*p* < 0.05) (Figure 3B,C). These findings indicate that moderate highland barley supplementation positively impacts skeletal muscle health and functionality in mice. However, as the supplementation level increased, the quadriceps muscle organ index returned to normal levels. Notably, the gastrocnemius muscle organ index in the HB60 group (8.70 ± 0.31) was lower than that in the HB20 group (10.02 ± 0.36) by approximately 13.15%, and this difference was significant (*p* < 0.05). This suggests that high doses of highland barley supplementation may lead to a decline in fatigue resistance, which may be related to the reduction in muscle index.

### 3.3. Effects of Different Levels of Highland Barley Intake on the Gut Microbiota of Mice

**Figure 4:**
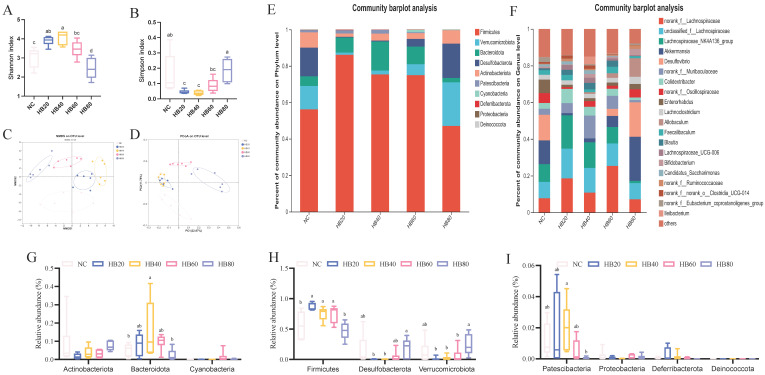
Highland barley supplementation altered the composition of the gut microbiota in mice (*n* = 6). (**A**) Shannon index, (**B**) Simpson index, (**C**,**D**) beta diversity, (**E**) relative abundance at the phylum level, (**F**) relative abundance at the genus level, (**G**) Actinobacteriota, Bacteroidota, Cyanobacteria, (**H**) Firmicutes, Desulfobacterota, Verrucomicrobiota, and (**I**) Patescibacteria, Proteobacteria, Deferribacterota, Deinococcota. Note: Bars with different letters (e.g., a, b, c) indicate significant differences at *p* < 0.05. Bars without letters indicate no significant difference.

Diet has a significant impact on the structure of the gut microbiota. Previous studies have identified genera such as *Turicibacter* and *unclassified Lachnospiraceae* as being associated with the body’s resistance to fatigue [19,20]. To investigate this further, we conducted 16S rRNA gene sequencing on fecal samples. The Shannon index in the HB20 group was significantly higher than that in the NC group, with a difference of 28.69% (*p* < 0.05). However, as the amount of highland supplementation increased, the Shannon index gradually decreased. The Shannon index was 19% lower in the HB80 group than in the NC group, and this difference was significant (*p* < 0.05). On the other hand, the Simpson index in the HB20 group was 72% lower than that in the NC group, and this difference was significant (*p* < 0.05), but this value gradually returned to levels similar to that of the NC group as highland barley intake increased (Figure 4A,B). PCoA plots were used to visualize β-diversity, representing the composition of the gut microbiota across the different dietary groups (Figure 4C,D). As the highland barley intake increased, the clusters of each group showed varying degrees of shift. This suggests that the supplementation with highland barley altered the composition of the gut microbiota in mice.

As shown in Figure 4, supplementation with highland barley significantly altered the relative abundances of the phyla *Bacteroidota*, *Firmicutes*, *Desulfobacterota*, *Verrucomicrobiota*, and *Patescibacteria* (Figure 4E). The HB20 group exhibited the highest relative abundance of Firmicutes. This value decreased as the intake amount increased, though in all supplemented groups, it remained significantly higher than that of the NC group (*p* < 0.05). The relative abundance of Firmicutes in the HB80 group was lower than that in the NC group, but no significant difference was observed (Figure 4H). Additionally, the relative abundances of Desulfobacterota and Verrucomicrobiota were higher in the HB80 group compared to the NC group, whereas no significant differences were observed in other supplementation groups regarding these phyla (Figure 4H).

At the genus level, the relative abundances of several genera, including *Lachnospiraceae_NK4A136_group*, *norank_f__Muribaculaceae*, *Allobaculum*, *unclassified_f__Lachnospiraceae*, *Akkermansia*, *Desulfovibrio*, Lachnospiraceae_UCG-006, *Candidatus_Saccharimonas*, *Turicibacter*, *norank_f__Peptococcaceae*, *norank_f__norank_o__RF39*, *unclassified_f__Ruminococcaceae*, *norank_f__Lachnospiraceae*, *Colidextribacter*, *Blautia*, and *norank_f__Eubacterium_coprostanoligenes_group* were altered by the highland barley supplementation (Figure 4F).

**Figure 5:**
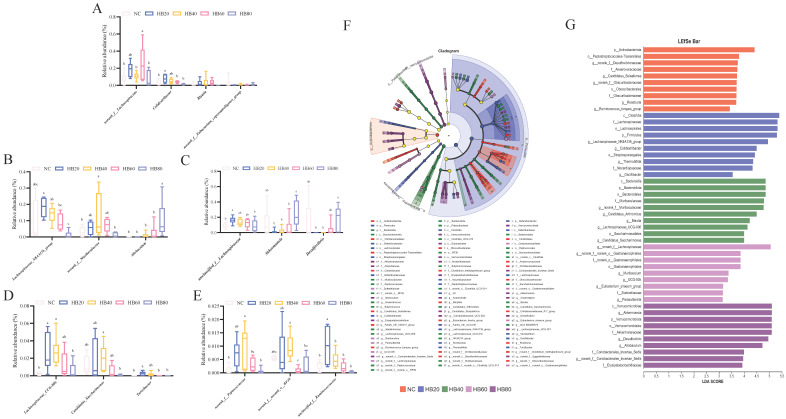
(**A**) *Lachnospiraceae_NK4A136_group*, *norank_f__Muribaculaceae*, *Allobaculum*; (**B**) *unclassified_f__Lachnospiraceae*, *Akkermansia*, *Desulfovibrio*; (**C**) *Lachnospiraceae_UCG-006*, *Candidatus_Saccharimonas*, *Turicibacter*; (**D**) *norank_f__Peptococcaceae*, *norank_f__norank_o__RF39*, *unclassified_f__Ruminococcaceae*; (**E**) *norank_f__Lachnospiraceae*, *Colidextribacter*, *Blautia*, *norank_f__Eubacterium_coprostanoligenes_group*; (**F**) Phylogenetic tree showing the relative abundance of gut microbiota in all groups, with circles representing the phylogenetic levels from phylum (innermost circle) to species (outermost circle). The diameter of each circle is proportional to the abundance of the taxonomic unit; (**G**) Linear discriminant analysis (LDA) effect size (LEfSe) comparison of gut microbiota in each group with an LDA score > 3. Note: Bars with different letters (e.g., a, b, c) indicate significant differences at *p* < 0.05. Bars without letters indicate no significant difference.

Further comparisons of the abundance of different microbiota between groups (Figure 5) showed that, compared to the NC group, the HB20 group exhibited a significant increase in the abundance of *unclassified_f_Lachnospiraceae*, *Lachnospiraceae_UCG-006*, *Turicibacter*, *unclassified_f_Ruminococcaceae*, and *Colidextribacter* (*p* < 0.05). In the HB40 group, significantly higher abundance was observed for *norank_f_Muribaculaceae*, *Lachnospiraceae_UCG-006*, and *norank_f_Peptococcaceae* (*p* < 0.05) (Figure 5C). Additionally, the HB60 group had significantly higher abundance of *norank_f_Lachnospiraceae* (Figure 5B) (*p* < 0.05), while the HB80 group showed significantly increased abundance of *Allobaculum* (Figure 5A) (*p* < 0.05).

Compared to the HB20 group, the HB80 group showed lower abundances of *Lachnospiraceae_NK4A136_group*, *unclassified_f_Lachnospiraceae*, *Lachnospiraceae_UCG-006*, *Turicibacter*, and *Colidextribacter*, but higher abundances of *Allobaculum*, *Akkermansia*, and *Desulfovibrio* (*p* < 0.05) (Figure 5A). The relative abundances of *Lachnospiraceae_NK4A136_group*, *Turicibacter*, *norank_f__norank_o__RF39*, *unclassified_f__Ruminococcaceae*, and *Colidextribacter* were lower in the HB60 group compared to the HB20 group (*p* < 0.05), with no significant differences in the overall microbial abundance (Figure 5D). Additionally, the abundance of *Lachnospiraceae_NK4A136_group* and *unclassified_f__Ruminococcaceae* decreased with higher highland barley supplementation, while that of *Allobaculum* and *Akkermansia* increased with increasing supplementation levels.

Moreover, LEfSe analysis from the phylum to the genus level was used to identify specific bacterial groups, with a logarithmic LDA score threshold of 3 used to determine the distinguishing features of each group (Figure 5F,G). As shown in Figure 5G, the HB20 group was enriched in *Lachnospiraceae_NK4A136_group*, *Colidextribacter*, *Thermobifida*, *unclassified_f__Ruminococcaceae*, *Oscillibacter*, and *Romboutsia*. The HB40 group was enriched in *norank_f__Muribaculaceae*, *Candidatus_Arthromitus*, *Blautia*, *Lachnospiraceae_UCG-006*, *Candidatus_Saccharimonas*, and *norank_f__norank_o__Clostridia_UCG-014*. The HB60 group was enriched in *norank_f__Lachnospiraceae*, *norank_f__norank_o__Gastranaerophilales*, *unclassified_f__Erysipelotrichaceae*, *Muribaculum*, *UCG-005*, and *Eubacterium_siraeum_group*. Additionally, the HB80 group was enriched in *Akkermansia* and *Desulfovibrio* and showed higher abundance of *Allobaculum*, *norank_f__Coriobacteriales_Incertae_Sedis*, and *Anaerostipes*. This corresponds with the aforementioned changes in microbiota abundance (Figure 5A,E).

Highland barley intake not only significantly affected the organ index and gut microbiota, but may also influence the host’s energy metabolism, accumulation of metabolic waste, and oxidative stress levels through modulation of the gut microbiota. Therefore, the next section of this study analyzes the specific effects of different highland barley intake levels on these metabolic indicators.

### 3.4. Effects of Different Levels of Highland Barley Intake on Energy Metabolism in Mice

During exercise, the body relies on glucose, liver glycogen, and muscle glycogen to maintain its energy supply and cellular homeostasis through ATP generation [41] and Na^+^-K^+^-ATPase activity, which support sustained exercise [42].

**Figure 6:**
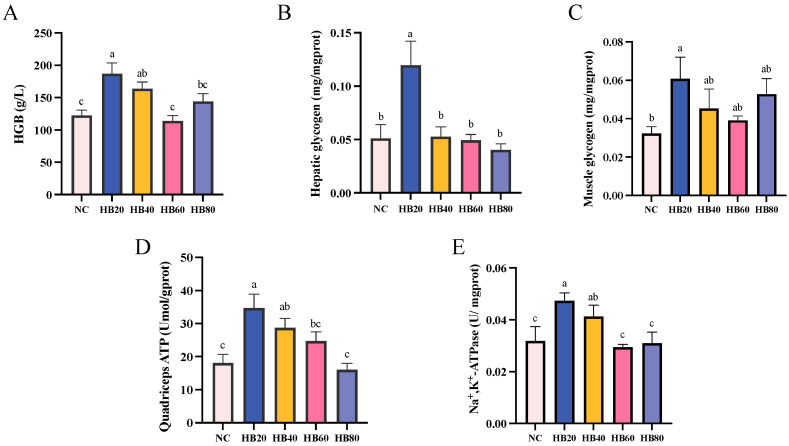
Effect of highland barley supplementation on HGB (**A**), HG (**B**), LG (**C**), ATP content in the quadriceps muscle (**D**), and Na^+^-K^+^-ATPase activity (**E**) in mice. Note: Bars with different letters (e.g., a, b, c) indicate significant differences at *p* < 0.05. Bars without letters indicate no significant difference.

Compared to the NC group, the HB20 group showed significantly increased levels of liver glycogen (0.12 ± 0.02) and muscle glycogen (0.06 ± 0.01), with differences of 134.48% and 87.75%, respectively (*p* < 0.05) (Figure 6B,C). Additionally, this group showed increased levels of HGB, ATP content, and Na^+^-K^+^-ATPase activity, with increases of 52.65%, 92.07%, and 48.39%, respectively (*p* < 0.05) (Figure 6A,D,E). However, as the level of supplementation with highland barley increased, liver and muscle glycogen levels decreased; the glycogen storage levels in the HB60 and HB80 groups approached those of the NC group. Excessive supplementation with highland barley did not further enhance the level of HGB, ATP content, or Na^+^-K^+^-ATPase activity. In fact, when the highland barley supplementation reached 60% or even 80%, the HGB levels, ATP content, and Na^+^-K^+^-ATPase activity levels in the HB60 and HB80 groups were significantly lower than those in the HB20 group (*p* < 0.05).

### 3.5. Effect of Different Levels of Highland Barley Supplementation on Accumulation of Metabolic Waste in Mice

During exercise, the body accelerates glycolysis and protein metabolism, producing lactate and nitrogen-containing compounds (such as BUN). The accumulation of these metabolic wastes can lead to limited energy supply and muscle fatigue [43].

**Figure 7:**
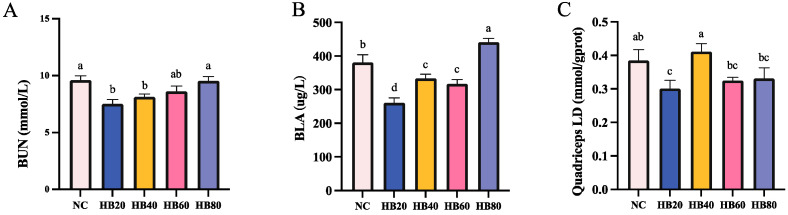
Effect of highland barley supplementation on BUN (**A**), BLA (**B**), and LD (**C**) levels in mice. Note: Bars with different letters (e.g., a, b, c, d) indicate significant differences at *p* < 0.05. Bars without letters indicate no significant difference.

With changes in highland barley intake, both the metabolic rate and the waste clearance rate in the body were affected, as evidenced by the changes in the levels of indicators of metabolic waste accumulation in the mice. After exhaustive swimming, the HB20 group showed significantly reduced BLA (31.52%), quadriceps LD (21.83%), and BUN (21.72%) levels compared to the NC group (*p* < 0.05) (Figure 7A–C). In contrast, the HB80 group had significantly higher BLA (441.43 ± 11.42) than either the NC group (15.79%) or the HB20 group (69.10%) (*p* < 0.05). The BUN (9.55 ± 0.36) level in the HB80 group was also 26.91% higher than that in the HB20 group, and this difference was significant (*p* < 0.05) (Figure 7A).

### 3.6. Effect of Different Levels of Highland Barley Supplementation on Oxidative Stress in Mice

Exercise induces the generation of ROS, leading to lipid peroxidation and an increase in oxidative stress levels. Antioxidant enzymes such as CAT, SOD, and GSH-Px mitigate exercise-induced tissue damage and exert anti-fatigue effects by enhancing antioxidant defenses [44].

**Figure 8:**
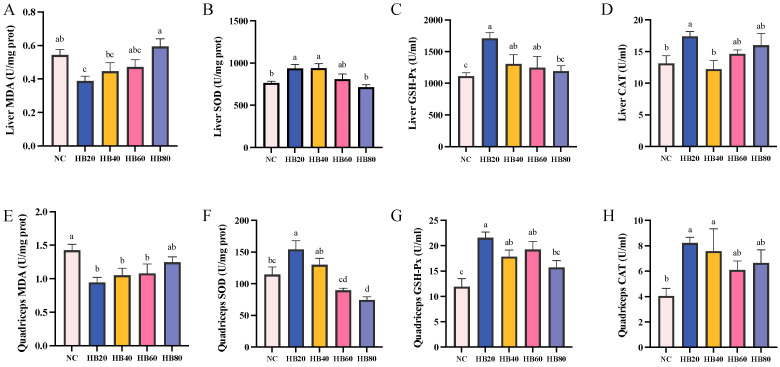
Effect of highland barley supplementation on MDA (**A**), SOD (**B**), GSH-Px (**C**), and CAT (**D**) levels in the liver and on MDA (**E**), SOD (**F**), GSH-Px (**G**), and CAT (**H**) levels in the quadriceps muscle. Note: Bars with different letters (e.g., a, b, c, d) indicate significant differences at *p* < 0.05. Bars without letters indicate no significant difference.

Compared to the NC group, the HB20 group had significantly higher liver and quadriceps SOD, GSH-Px, and CAT levels: 34.55%, 81.14%, and 103.31% higher, respectively (*p* < 0.05). Meanwhile, the liver and quadriceps MDA levels in the HB20 group were significantly reduced, being 28.63% and 33.76% lower (*p* < 0.05). In the HB80 group, the levels of liver and quadriceps SOD, as well as of liver and quadriceps GSH-Px, were lower than those in the HB20 group (*p* < 0.05). Additionally, the liver MDA levels in the HB80 group were 53.2% higher than those in the HB20 group, and this difference was significant (*p* < 0.05) (Figure 8).

## 4. Discussion

The effects of varying doses of highland barley supplementation on the anti-fatigue capacity of mice were examined in this study. Our results show that moderate supplementation (20% of total dietary energy) significantly enhances exercise endurance and reduces oxidative stress, primarily by modulating energy metabolism and the composition of the gut microbiota. These findings highlight highland barley’s potential as a functional food to combat fatigue. However, excessive supplementation (≥60%) led to diminished benefits, emphasizing the need for optimizing dosage in practical applications. These findings provide valuable insights for future studies of dietary supplementation aimed at determining optimal levels of barley intake for the general population and offer a reference for the development of anti-fatigue products by industry.

Exercise endurance is a widely recognized indicator of anti-fatigue effects [45]. As physical exertion increases, the body utilizes glycogen stores in the liver and muscles to sustain energy production [41]. This finding is in agreement with those of previous research, which has highlighted the role of polysaccharides in glycogen storage and energy metabolism during exercise [46]. For instance, studies have shown that okra polysaccharides enhance muscle and liver glycogen reserves by 16.66–58.69% and muscle ATP content by approximately 46.22% in mice [16,46]. Similarly, Xu et al. reported that oral administration of β-glucan (25, 50, and 100 mg/kg/day) extended the exhaustive swimming time of mice by 23.03%, 63.50%, and 89.18%, respectively. These effects were attributed to β-glucan’s influence on energy metabolism, particularly its effects of increasing muscle glycogen levels (28.42%) and enhancing SOD antioxidant enzyme activity by approximately 27.78% [47]. Furthermore, another study demonstrated that β-glucan supplementation (312.5 mg/kg/day) extended the exhaustive running time of rats by 71.8 ± 4.8 min, and this difference was significant. The authors suggested that this outcome could be due to β-glucan’s role in improving fatty acid function, reducing carbohydrate consumption, decreasing glycogen depletion, and enhancing liver glycogen storage, which collectively contribute to improved metabolic control and energy metabolism activation during exercise [16]. In our study, highland barley, which contains 2.13 g/100 g of β-glucan (Table 1), was used to supplement the diet of mice at 20%. This resulted in an intake of approximately 447.5 mg/kg of β-glucan, which exceeds the β-glucan intake levels used in previous studies [16]. The supplementation with highland barley at 20% significantly enhanced exercise endurance in mice, as evidenced by a 92.64% increase in exhaustive swimming time and a 69.51% increase in running time (Figure 3A). Additionally, muscle glycogen content increased by 87.75% and SOD antioxidant enzyme activity was enhanced by approximately 34.55%. These findings are consistent with those of earlier studies on β-glucan [16,47], supporting the notion that β-glucan supplementation positively affects energy metabolism, thereby improving exercise endurance.

During exercise, the body increases its energy supply by accelerating glycolysis. Anaerobic metabolism breaks down glucose, causing blood lactate (BLA) and muscle lactate (LD) accumulation [48]. When carbohydrate and lipid stores are low, protein metabolism is triggered, producing nitrogen compounds like blood urea nitrogen (BUN) [43]. These byproducts, lactate and BUN, are linked to fatigue because they impair energy production and muscle function. In a previous study, Gao et al. demonstrated that polysaccharides derived from okra, when administered at doses of 50, 100, and 200 mg/kg, reduced lactate accumulation by approximately 33.33% and improved endurance by enhancing glycogen reserves by 16.66–58.69% [46]. Xu et al. also reported that β-glucan supplementation extended endurance by modulating energy metabolism, notably increasing liver glycogen by 25% and decreasing metabolic waste accumulation, with the latter effect including a significant reduction in BUN levels by 24.1% [16]. These findings highlight the importance of targeting metabolic waste products to alleviate fatigue. Our study demonstrates that 20% highland barley supplementation significantly reduces the accumulation of metabolic byproducts after an exhaustive swimming test. Specifically, HB20 supplementation reduced blood lactate levels by 31.52%, muscle lactate by 21.83%, and serum urea nitrogen by 21.72% (Figure 6). This suggests that highland barley may exert a protective effect by attenuating the accumulation of these fatigue-associated metabolites, potentially through its antioxidant and anti-inflammatory properties. Furthermore, the magnitude of the reduction in lactate and urea nitrogen levels observed in our study is comparable to that reported by Gao et al. [46] and Xu et al. [16], indicating that highland barley could be a promising dietary supplement for improving endurance and mitigating fatigue.

The rapid removal of metabolic waste is critical in preventing fatigue. During exercise, reactive oxygen species (ROS) are generated as part of the body’s response to repeated stress [8,9]. However, prolonged physical exertion can result in excessive ROS, leading to lipid peroxidation and disruptions in metabolic processes, which further contribute to the accumulation of metabolic waste [44]. Malondialdehyde (MDA), a byproduct of lipid peroxidation, serves as a common marker for oxidative stress [49]. ROS also impair ATP production, exacerbating fatigue [50]. Antioxidants such as catalase (CAT), superoxide dismutase (SOD), and glutathione peroxidase (GSH-Px) help mitigate ROS, reduce oxidative stress, and improve anti-fatigue capacity [44,51]. Previous studies have demonstrated that polysaccharides can decrease levels of metabolic waste markers like blood lactate (BLA) and blood urea nitrogen (BUN), primarily due to their antioxidant effects [52]. Similarly, proteins and peptides have been shown to enhance exercise performance and accelerate fatigue recovery by improving energy supply and bolstering antioxidant defenses [53]. For instance, Li et al. reported that a 225 mg/kg dose of ginseng peptides significantly increased swimming endurance by 35.24%, elevated SOD levels by 13.54%, and reduced post-exercise lactate and BUN levels by approximately 27.87%, thereby alleviating oxidative stress and fatigue [54]. Polyphenols, which are natural antioxidants, also exhibit potent free radical-scavenging abilities and have been linked to anti-fatigue effects. Teng et al. found that tea polyphenols, administered at doses of 50, 100, and 200 mg/kg, increased SOD activity by roughly 33.33%, reduced oxidative stress, and extended swimming time by about 46.67% [25]. In our study, the daily intake of highland barley by the mice was approximately 630 mg/kg tyrosine, 779.9 mg/kg phenylalanine, and 69.3 mg/kg polyphenols (Table 1), which closely mirrors the dosages used in Teng et al.’s study [25]. In contrast, our results showed that 20% highland barley supplementation increased swimming time by 92.57% (Figure 2C), enhanced SOD levels by 34.55%, and reduced BUN levels by 21.72% (Figure 8). These findings suggest that highland barley supplementation not only alleviates oxidative stress but also significantly boosts anti-fatigue capacity. This aligns with the results of Li et al. [54] and underscores the potential of highland barley to enhance exercise performance and promote energy metabolism through its antioxidant properties.

Research has shown that the enrichment of *Turicibacter* can regulate muscle function and alleviate fatigue through its participation in various amino acid-metabolism pathways, organic acid-metabolism pathways, and pyrimidine-metabolism pathways [18,19,20]. Additionally, *Lachnospiraceae NK4A136* can exert anti-fatigue effects by promoting the catabolism of nutrients [21]. Our study shows that the supplementation with 20% highland barley significantly enriched the abundance of *Turicibacter* and *Lachnospiraceae NK4A136* in fatigued mice (Figure 5A). Moreover, *Lachnospiraceae_UCG-006* has been reported to improve the body’s oxidative stress state [55]. In our study, the 20% highland barley supplementation significantly increased the abundance of *Lachnospiraceae_UCG-006* in fatigued mice (Figure 5C). It has been reported that polysaccharides are key substances for the enrichment of *Lachnospiraceae_UCG-006* [56]. Furthermore, Zang et al. pointed out that highland barley β-glucan can increase the abundance of *Lachnospiraceae_UCG-006* [57]. Therefore, the polysaccharides in 20% highland barley, especially β-glucan, significantly enrich the abundance of *Lachnospiraceae_UCG-006*, enhancing the activity of endogenous antioxidant enzymes, alleviating oxidative stress induced by fatigue, and exerting anti-fatigue effects. In conclusion, the 20% highland barley supplementation regulates the balance of gut microbiota, improves energy metabolism, reduces the accumulation of metabolic waste products caused by fatigue, and enhances the body’s resistance to fatigue.

However, studies have also found that as the amount of highland barley in the diet increases, there is no clear dose-response relationship for improved anti-fatigue effects. In food or nutrient intake, the dose is a key factor. Within an appropriate intake range, certain components may exert beneficial effects, but when the intake becomes excessive, these components may no longer produce enhanced benefits and may even lead to negative effects [58,59]. Our study shows that as the intake of highland barley increases, the anti-fatigue capacity of mice does not continue to improve. Excessive intake of certain components in highland barley, such as β-glucan and some anti-nutritional factors, may interfere with the absorption and effective utilization of energy components, limiting the efficient intake of energy. At the same time, the abundant β-glucan and polyphenols in highland barley, while contributing to enhanced anti-fatigue effects, may reduce energy utilization when consumed in excessive amounts (60% or 80% of the total diet) by inhibiting α-amylase activity and delaying starch digestion [60]. Additionally, anti-nutritional factors such as lectins and phytic acid in highland barley may hinder nutrient absorption when consumed excessively [61]. The high iron content in highland barley may also enhance oxidative stress, further affecting energy metabolism and preventing the clearance of metabolic waste products [62,63]. Furthermore, excessive highland barley intake could inhibit the absorption of essential energy components (such as proteins and carbohydrates), affecting their normal effects on metabolism and biological systems and preventing the expected anti-fatigue results. For example, the gluten structure in highland barley proteins is dense, leading to a longer digestion time, and excessive intake may cause incomplete digestion, thereby affecting energy replenishment [64]. Additionally, highland barley contains a high amount of large starch granules with lower digestibility, which could limit energy supply [65]. From a gut microbiota perspective, excessive intake of highland barley may lead to the proliferation of harmful bacteria, such as *Desulfovibrio* (sulfate-reducing bacteria), which tend to colonize the gut of fatigued individuals [66], and *Allobaculum*, which is associated with intestinal epithelial inflammation and colitis [67]. It could also reduce the abundance of beneficial bacteria, such as *Deferribacteres* [68] and *Colidextribacter* [69]. On the other hand, in our study, the *Akkermansia* abundance in the HB80 group was significantly higher than that in the HB20 group (Figure 5B). *Akkermansia* is commonly reported to have beneficial effects, such as weight reduction and decreased lipid accumulation, and is negatively correlated with body weight [70]. On the other hand, mice with a lower-than-normal body weight typically perform worse in studies of exercise performance [71]. Our study results are consistent with these findings.

In summary, excessive intake of highland barley may compromise its anti-fatigue benefits by affecting starch and protein digestion and the composition of the gut microbiota. Therefore, determining an optimal intake level is essential. While moderate consumption enhances endurance, excessive intake may lead to adverse effects, ultimately diminishing its potential benefits. However, the study’s relatively small sample size may affect statistical power, and future research will address this by conducting power calculations and increasing sample sizes for greater reliability. Additionally, as this study was conducted in an animal model, the results may not fully translate to humans due to differences in metabolism and gut microbiota. To address this, clinical trials will be conducted to validate the findings and assess their relevance to human health. Although key physiological mechanisms were explored, further investigation is needed to elucidate the underlying molecular pathways, particularly those related to mitochondrial function and muscle-fiber composition. These efforts will ultimately help translate the findings into dietary guidelines that increase health benefits and reduce risks associated with excessive intake.

## 5. Conclusions

This study concludes that a 20% intake of highland barley significantly enhances anti-fatigue capacity by regulating oxidative stress, promoting energy metabolism, and altering the composition of the gut microbiota. The increase in antioxidant-enzyme activity reduces metabolic waste and supports muscle growth, while the proliferation of beneficial gut bacteria positively correlates with endurance. However, higher doses (60–80%) may disrupt nutrient absorption and reduce energy reserves. Future research should address sample-size limitations, validate these findings in clinical trials, and investigate the molecular mechanisms behind the observed effects.

## Figures and Tables

**Table 1:** Basic nutrient content and amino acid content of highland barley powder.

Component	Content (g/100 g)	Amino Acid	Content (g/100 g)
Carbohydrates	62.2	Aspartic Acid	6.228 ± 2.9
Protein	12.7	Glutamic Acid	16.033 ± 3.0
Fat	3.1	Valine	3.063 ± 4.4
Dietary Fiber	8.89	Isoleucine	2.547 ± 1.6
Ash	1.8	Leucine	5.304 ± 2.1
Moisture	11.3	Tyrosine	2.993 ± 0.4
β-glucan	2.13	Phenylalanine	3.719 ± 0.7
Polyphenols	0.33	Lysine	3.906 ± 3.3
Crude Polysaccharides	9.74	Arginine	4.718 ± 2.3

## Data Availability

The original contributions presented in the study are included in the article/Supplementary Materials, further inquiries can be directed to the corresponding author.

## Supplementary Materials

The following supporting information can be downloaded at: https://www.mdpi.com/article/10.3390/nu17040733/s1, Table S1. Energy ratio provided by nutrients (%). Table S2. Composition of experimental diets.

## Abbreviations

The following abbreviations are used in this manuscript:

**Acronym**	**Full Name**
BUN	Blood urea nitrogen
LD	Lactic acid
HG	Hepatic glycogen
MG	Muscle glycogen
SOD	Superoxide dismutase
MDA	Malondialdehyde
GSH-Px	Glutathione peroxidase
CAT	Catalase
ROS	Reactive oxygen species
