# Supplementary material for: Moderate Highland Barley Intake Affects Anti-Fatigue Capacity in Mice via Metabolism, Anti-Oxidative Effects and Gut Microbiota
Source: Nutrients. 2025 Feb 19;17(4):733. doi: 10.3390/nu17040733 (PMC11858136; doi:10.3390/nu17040733)
Supplement: Supplementary file 1 [file nutrients-17-00733-s001.zip › nutrients-3470095-supplementary.pdf]

Supplementary Materials:

Table S1. Energy ratio provided by nutrients（%）.

Ingredient	Control Group	Intervention Group			
	（NC）	HB20	HB40	HB60	HB80
Protein	20	20	20	20	20
Carbohydrate	70	20	20	20	20
Fat	10	60	60	60	60
Total			100		

Table S2. Composition of experimental diets.

	NC		HB20		HB40		HB60		HB80	
	g%	kcal%	g%	kcal%	g%	kcal%	g%	kcal%	g%	kcal%
Highland barley	0	0	230.15	753.83	460.25	1507.48	690.46	2261.48	920.61	3015.29
Casein, 30 Mesh	140	560	110.77	443.08	81.54	326.16	52.3	209.2	23.07	92.28
L-Cystine	1.8	7.2	1.8	7.2	1.80	7.20	1.8	7.2	1.80	7.20
Corn Starch	495.69	1983	352.52	1410.09	209.38	837.53	66.182	264.728	0.00	0.00
Maltodextrin 10	125	500	125	500	125.00	500.00	125	500	48.01	192.05
Sucrose	100	400	100	400	100.00	400.00	100	400	100.00	400.00
Cellulose	50	0	29.54	0	9.08	0.00	0	0	0.00	0.00
Soybean Oil	40	360	32.86	295.74	25.73	231.57	18.59	167.31	11.46	103.14
t-Butylhydroquinone	0.01	0	0.01	0	0.01	0.00	0.008	0	0.01	0.00
Mineral Mix S10022M	35	0	35	0	35.00	0.00	35	0	35.00	0.00
Vitamin Mix V10037	10	40	10	40	10.00	40.00	10	40	10.00	40.00
Choline Bitartrate	2.5	0	2.5	0	2.50	0.00	2.5	0	2.50	0.00
Total	1 000.00	3850.2	1 030.15	3849.94	1060.29	3849.94	1101.84	3849.918	1152.46	3849.96
